# A case of squamous cell carcinoma of the skin due to the molecularly confirmed Lynch Syndrome

**DOI:** 10.1186/s13053-015-0033-2

**Published:** 2015-05-16

**Authors:** Steven Sorscher

**Affiliations:** Division of Oncology, Washington University in St. Louis, 660 S. Euclid Avenue CB 8056, St. Louis, MO USA

**Keywords:** Lynch, Squamous, Colon cancer

## Abstract

Patients with Lynch Syndrome are at high risk for developing a variety of cancers including cancers of the colon or rectum, small bowel, stomach, uterus, renal pelvis, ureter, biliary tract, ovaries, brain and pancreas (N Engl J Med 348: 919-32, 2003; Gut 57:1097-101, 2008; NCCN, Inc Guideline. Ft. Washington, PA. Online Version 2.2014). Lack of MLH-1 and MSH-2 expression commonly result from germline mutations in this inherited cancer syndrome. Here, we report the case of a patient with a molecularly confirmed germline mutation in MLH-1 along with a colon cancer showing lack of expression of MLH-1 as well as a squamous cell cancer of the skin from the abdominal wall also demonstrating lack of expression of MLH-1. This case appears to represent the second case report of a squamous cell skin cancer apparently due to the Lynch Syndrome and further supports a proposed relationship between Lynch Syndrome and these tumors.

## Background

Lynch Syndrome is the most common inherited colon cancer syndrome and also is associated with an increased risk for patients developing cancers of other tissue origin. It has only once been reportedly associated with squamous cell skin cancer.

## Case presentation

A 54-year-old man underwent a right hemicolectomy on February 10, 2011 and the pathology revealed a T4b (14 cm and invading the pericolic fat), N1a (1 of 31 lymph nodes involved with malignancy) M0 adenocarcinoma of the colon. There was no family history of colon or other gastrointestinal malignancies. CT scanning showed no radiographic evidence of distant disease. He received standard adjuvant chemotherapy with 12 doses of modified FOLFOX-6 chemotherapy (5-fluorouracil, leucovorin and oxaliplatin) and has had no recurrence to date.

The malignancy demonstrated loss of MLH-1 expression and no BRAF mutation was identified (wild-type (WT)BRAF). In January 2013, a routine follow-up abdominal MRI showed a new 1.8 × 2.2 × 2.3 hyperintense mass within the soft tissue on the left abdominal wall, just above the umbilicus. The mass was removed and identified as a “squamous cell carcinoma, moderately differentiated, invasive (3.7 cm)”, with no tumor present at peripheral or deep margins. Immunohistochemical (IHC) staining showed loss of expression of MLH-1 and PMS-2. The pathologist also performed IHC staining of the patient’s colonic adenocarcinoma which also showed “loss of MLH-1 expression within the tumor cells”. Normal controls were provided for both the colon cancer and squamous cell skin cancer as part of the IHC staining done.

The patient was seen by the medical genetics service and germline analysis revealed that the patient’s germline harbored a mutation in MLH-1, specifically the 1772del4 mutation. PCR-based full MLH-1 sequence and rearrangement analysis was performed using colaris testing for full sequence analysis (Myriad Genetics, Salt Lake City, UT). This confirmed a diagnosis of the patient having the Lynch Syndrome.

## Conclusions

The Lynch Syndrome is the most common inherited syndrome predisposing patients to developing colon cancer. The inherited syndrome is an autosomal dominant disorder characterized by a germline mutation in one of several different mismatch repair genes (most commonly MLH-1, MSH-2, MSH-6 or PMS-2). Germline mutations in MLH-1 comprise 32 % of cases of the Lynch Syndrome and result in tumors lacking MLH-1 expression, as seen in our patient’s colon cancer [1]–[3]. For those with germline mutations in MLH-1, lifetime risks of cancers include colon (40–80 %), endometrial (25–60 %), stomach (1–13 %), ovary (4–24 %), hepatobiliary (1.4–4 %), urinary tract (1–4 %), small bowel (3–6 %), brain (1–3 %), pancreas (1–6 %) and sebaceous neoplasms (1–9 %) [3]. Lack of expression of MLH-1 by IHC can also result from a somatic cell mutation in BRAF. However, this patient’s colon cancer showed WT BRAF.

Our patient had two invasive malignancies, both demonstrating lack of MLH-1 expression and genetic testing verified a germline mutation in the MLH-1 gene, confirming a molecular diagnosis of the Lynch Syndrome. Sporadic squamous cell skin cancers have not been studied extensively for MLH-1 expression; one small series suggests these tumors infrequently lack MLH-1 expression [4]. Thus, it remains possible that this patient’s skin cancer was sporadic . However, given the lack of expression of MLH-1 in the colon cancer and the corresponding germline mutation, it would seem far more likely that this patient’s squamous cell carcinoma was related to the Lynch syndrome. One other case of true squamous cell cancer of the skin in a patient with the Lynch Syndrome has been reported [5].

Because squamous cell skin cancer is relatively common, considering the possibility that it is related to the Lynch Syndrome in a particular patient should probably be reserved for patients highly suspected of having Lynch Syndrome or those with known Lynch Syndrome. This second report of a patient with the Lynch Syndrome and a squamous cell skin cancer lacking MLH-1 expression supports the conclusions of the authors of the first case report that, although rare, inheritance of a germline mutation in MLH-1 might also be considered a risk factor for developing squamous cell skin cancer.

## Consent statement

Written informed consent was obtained from the patient for publication of this Case Report and any accompanying images. A copy of the written consent is available for review by the Editor-in-Chief of this journal.
